# Bioinformatics-Based Analysis: Noncoding RNA-Mediated COL10A1 Is Associated with Poor Prognosis and Immune Cell Infiltration in Pancreatic Cancer

**DOI:** 10.1155/2022/7904982

**Published:** 2022-09-05

**Authors:** Qi Liu, Hongyu Zhao, Yu Guo, Kai Zhang, Fengjia Shang, Tongjun Liu

**Affiliations:** ^1^Department of Colorectal Surgery, the Second Hospital of Jilin University, Changchun 130041, China; ^2^Department of Gastroenterology and Center of Digestive Endoscopy, the Second Hospital of Jilin University, Changchun 130041, China

## Abstract

**Background:**

Collagen type X alpha 1 (COL10A1) is a structural component of the extracellular matrix that is aberrantly expressed in a variety of cancer tissues. However, its role in pancreatic cancer progression is not well understood.

**Methods:**

The Cancer Genome Atlas (TCGA), Gene Expression Omnibus (GEO), and Gene Expression Profiling Interaction Analysis (GEPIA) data were employed to explore the expression of COL10A1 in normal and tumor tissues and its prognostic value in pancreatic adenocarcinoma. The clinical data of pancreatic cancer in TCGA were used to explore the relationship between COL10A1 and clinical features. Genes coexpressed with COL10A1 were explored using multiple databases and analyzed for functional enrichment. In addition, the lncRNA/miRNA/COL10A1 axis that may be involved in COL10A1 regulation in pancreatic cancer was explored by constructing a competitive endogenous RNA (ceRNA) regulatory axis. Finally, COL10A1 was analyzed for correlation with immune cell infiltration and various immune checkpoint molecules in pancreatic cancer.

**Results:**

It was found that the expression of COL10A1 was significantly increased in pancreatic cancer tissues. High expression of COL10A1 was related to the clinicopathological characteristics and the worse prognosis of pancreatic cancer patients. The TUG1/miR-144-3p/COL10A1 axis was identified as the most likely upstream noncoding RNA pathway for COL10A1 in pancreatic cancer. Besides, in pancreatic adenocarcinoma, the expression level of COL10A1 showed a significant positive correlation with tumor immune cell infiltration, biomarkers of immune cells, and expression of immune checkpoint molecules.

**Conclusion:**

COL10A1 is an early diagnostic marker, and its high expression correlates with immune infiltration in pancreatic cancer. The TUG1/miR-144-3p/COL10A1 axis was identified as the most likely upstream noncoding RNA pathway for COL10A1 in pancreatic cancer.

## 1. Introduction

Pancreatic cancer (PAAD) is one of the deadliest malignancies. The early symptoms of PAAD are very insidious, and most of them are advanced at the time of clinical diagnosis [1]. The five-year survival rate after diagnosis of PAAD is about 10% [2]. Given the difficulty of early diagnosis of PAAD, it is particularly important to develop early diagnostic markers for PAAD. In addition, drugs based on tumor molecular pathogenesis, such as targeted therapy and immune checkpoint inhibitors, have become a new development direction for tumor therapy [3, 4]. In recent years, many studies on the molecular pathogenesis of PAAD have advanced the understanding of PAAD progression and provided potential targets for targeted therapy of PAAD [5]. However, there is currently no molecular subtyping based on immune signatures to facilitate a better understanding of the molecular mechanisms of PAAD, leading to early diagnosis and effective treatment [6].

Collagen X a-1 (collagen X) is a member of the collagen family, in which the COL10A1 gene encodes collagen involved in the formation of the extracellular matrix [7]. Recent studies have found that the overexpression of COL10A1 enhances the epithelial-mesenchymal transition of tumors and promotes tumor aggressiveness and disease progression [8]. Previous studies have shown that COL10A1 is significantly overexpressed in patients with colon cancer, gastric cancer, and breast cancer, and is associated with poor prognosis [9–11]. COL10A1 is poorly expressed in a variety of normal tissues, suggesting the potential utility of this gene as a diagnostic marker and therapeutic target for cancers [12]. However, the role of aberrant COL10A1 expression in the development and progression of PAAD and its molecular mechanisms remain unclear.

The tumor microenvironment (TME) refers to the close relationship between tumorigenesis, growth, and metastasis, and the formation of the internal and external environment in which tumor cells are located [13]. The presence of a TME allows tumor cells to alter and maintain the conditions for their survival and development through autocrine and paracrine secretion, promoting tumor growth and development [14, 15]. TME is composed of tumor cells, stromal cells, fibroblasts, and immune cells, and almost all immune cells are involved in tumor progression [16]. Tumor-infiltrating immune cells in TME can affect tumor cell progression directly or indirectly by inducing host immune responses, including the release of cytokines, cytokine receptors, or other factors [17]. For example, neutrophils are involved in immunosuppression and promote tumor progression [18]. Immune checkpoint blockade therapy revitalizes antitumor immunity by exploiting immune cell infiltration in the tumor [19]. Previous studies have shown that COL10A1 is specifically expressed in the tumor microenvironment and is associated with tumor angiogenesis [12]. However, whether COL10A1 is involved in the immune infiltration of PAAD has not been determined. Immunotherapy has shown significant efficacy in lung and breast cancers, but immunotherapy has not been applied to PAAD [20–22]. The efficiency of immunotherapies relies on an immunogenic TME [23]. In the immunosuppressive microenvironment, poor T-cell infiltration gives immune privilege to PAAD and affects the efficacy of immunotherapy in PAAD[24]. Promisingly, multiple studies have shown that immunotherapy can benefit some PAAD patients [25, 26]. Therefore, there is a need to explore the molecular mechanisms related to the immune signature of PAAD.

In this study, we investigated the differential expression of COL10A1 in normal and tumor tissues and its prognostic value in PAAD using TCGA, the GEPIA, and the GEO databases. Furthermore, upstream microRNAs (miRNAs) and long noncoding RNAs (lncRNAs) of COL10A1 were investigated by hypothesizing a competing endogenous RNA (ceRNA) regulatory axis. COL10A1 was analyzed for correlation with immune cell infiltration and various immune checkpoint molecules in pancreatic cancer, and the possible mechanisms affecting the prognosis were also discussed. The results of this study suggest that COL10A1 may influence the prognosis of cancer patients through its interaction with infiltrating immune cells.

## 2. Materials and Methods

### 2.1. Data Source

RNA-sequencing data and clinicopathological information from The Cancer Genome Atlas (TCGA) pancreatic adenocarcinoma patients were included in the study (https://genomecancer.ucsc.edu/). Data on RNA-Seq expression and matched clinicopathological information of 178 PAAD patients and 4 normal tissues adjacent to the cancer were obtained by TCGA tool Cancer Browser. Due to the small amount of normal tissue data in TCGA database, we downloaded the uniformly normalized pan-cancer data (PANCAN, *N* = 19131, *G* = 60499) from the UCSC (https://xenabrowser.net/) database for TCGA and GTEx, and obtained a total of 33 cancers in TCGA including PAAD. Expression data and corresponding normal tissue data in GTEx were used to compare COL10A1 gene expression in PAAD and pan-cancer. In this study, the datasets GSE15471, GSE101448, GSE62165, GSE16515, GSE57495, and GSE62452 were downloaded from Gene Expression Omnibus (GEO) (https://www.ncbi.nlm.nih.gov/geo/). Our studies were all derived from publicly available data from TCGA and GEO databases, and therefore, there were no ethical issues.

### 2.2. Differential Expression Analysis

After retaining the samples with clinical information and removing duplicate samples, we further extracted the COL10A1 gene expression data in PAAD from TCGA database. After log2 transformation of the expression values, a comparison between the tumor group and the normal group was performed. COL10A1 gene expression data were extracted from five datasets (GSE15471, GSE101448, GSE62165, GSE16515, and GSE62452) from the GEO database and analyzed using the ggplot2 package of the *R* software. The criteria for selecting the datasets were as follows: (1) All were human pancreatic samples; (2) the datasets contained pancreatic cancer tumor and nontumor control samples; and (3) the number of samples was not less than 30. The ggplot2 package of the *R* (version 3.6.3) software was used to analyze gene expression differences.

### 2.3. Survival Analysis

The Kaplan–Meier plotter (http://kmplot.com/analysis/) allows survival analysis of genes or miRNAs for more than 20 cancer types, including PAAD. The effect of COL10A1 expression on overall survival (OS) and disease-free survival (DFS) was analyzed using the Kaplan–Meier plotter. Using the dataset GSE57495, the survival curves of COL10A1 in PAAD were plotted as an external validation of the prognostic characteristics of COL10A1. GSE57495 contains 63 pancreatic cancer samples with complete follow-up information (survival status and survival time). Survival curves were constructed using the Kaplan–Meier method, and prognostic differences between different COL10A1 expression groups were assessed using the log-rank test. The area under the curve (AUC) for 1-, 2-, and 3-year survival was obtained by ROC analysis to verify the accuracy of the survival curves constructed with GSE57495. ROC analysis was performed using the *R* software package pROC.

### 2.4. Upstream miRNA Prediction

To predict the upstream binding miRNAs of COL10A1, we selected five established online gene prediction programs including RNA22, miRmap, microT, miRDB, and TargetScan. Only miRNAs that were present in more than two of these programs at the same time were included in the next study. The final 12 candidate miRNAs were obtained. StarBase (http://starbase.sysu.edu.cn/) provides miRNA-mRNA and miRNA-lncRNA interaction networks supported by CLIP-Seq experiments [27]. By combining 13 functional genomic annotations, StarBase can predict miRNAs and lncRNAs from miRNA-mediated regulatory networks. According to the endogenous competing RNA (ceRNA) hypothesis, miRNAs and COL10A1 should be negatively correlated. Therefore, a correlation analysis was performed for the predicted 12 miRNAs. The miR-144-3p-COL10A1 correlation was analyzed in PAAD using StarBase. In addition, StarBase was used to analyze the differential expression of miR-144-3p in PAAD. We performed a survival analysis of miR-144-3p using the Kaplan–Meier plotter.

### 2.5. Upstream lncRNA Prediction

The candidate lncRNAs that may bind to miR-144-3p were predicted using StarBase. A total of 96 lncRNAs were visualized using the Cellscape software. According to the competitive endogenous RNA hypothesis, microRNAs are known to cause gene silencing by binding the messenger RNA (mRNA), while lncRNAs can increase gene expression by competitively binding microRNAs. Therefore, there should be a positive correlation between lncRNAs and mRNAs. The association between 96 lncRNAs and miR-144-3p and the correlation between COL10A1 and 96 lncRNA expressions in PAAD were investigated using StarBase. The lncRNAs that met the criteria of being negatively correlated with miR-144-3p expression and positively correlated with COL10A1 expression in PAAD were considered eligible while setting a *p* < 0.05 as statistically significant. Gene Expression Profiling Interaction Analysis (GEPIA) (http://gepia.cancer-pku.cn/index.html) was used to analyze the expressions of KCNQ1OT1, LINC00662, DUXAP8, and TUG1 genes in PAAD. TUG1 expression in PAAD was validated using PAAD data from TCGA and the corresponding normal data from GTEx. The prognosis of TUG1 in pancreatic cancer was plotted using the survival package in the *R* software. Since dataset GSE57495 does not contain normal controls for pancreatic cancer samples, we validated the expression and prognosis of TUG1 in pancreatic cancer using dataset GSE62452.

### 2.6. Immunoinfiltration Analysis

Based on tumor sample data in TCGA, Tumor Immunity Evaluation Resource (TIMER2.0 (comp-genomics.org)) applies computational methods such as the deconvolution method to estimate the abundance of six tumor-infiltrating immune subgroups [28]. TIMER2.0 detects the association between immune infiltration and gene expression in TME by RNA-Seq expression profiling. In addition, TIMER2.0 can generate scatter plots of different gene expression correlations in selected tumors [28]. In this study, the correlation between COL10A1 expression and the abundance of six tumor-infiltrating immune subpopulations in pancreatic cancer was calculated using TIMER2.0. The relationship between COL10A1 gene expression and tumor purity was also included in the study. The correlation between COL10A1 expression and the expression levels of immune checkpoints or other prognostic markers in PAAD was investigated using TIMER 2.0.

### 2.7. Functional Enrichment Analysis

In this study, we used the UALCAN (http://ualcan.path.uab.edu/index.html) and the GEPIA databases to screen for genes coexpressed with COL10A1, respectively (Supplementary Table 1). Afterward, the same coexpressed genes in both databases were screened, and further, functional enrichment analysis was performed (Supplement Figure 1; Supplementary Table 2). Metascape (http://metascape.org) allows enrichment analysis of biological pathways with rich gene annotation capabilities [29]. In this study, Metascape was used to perform a functional enrichment analysis of COL10A1 and its coexpressed genes. We selected the most statistically significant terms as a visual network atlas to further identify the relationships between terms. Then, we used the protein-protein interaction network structure analysis accompanying the Metascape online tool to identify potential protein complexes.

### 2.8. Statistical Analysis

The Mann–Whitney *U* test was used to analyze the differential expression of COL10A1 in PAAD and normal tissues. The relationship between COL10A1 expression and pancreatic cancer clinicopathology was analyzed by a univariate logistic regression analysis. Survival risk factors for PAAD were screened using the univariate Cox regression analysis. All studies were analyzed using the software *R* (version 3.6.3). Statistical significance was reflected by significance markers: ns, *p* ≥ 0.05; *p*^*∗*^ < 0.05; *p*^*∗∗*^ < 0.01; *p*^*∗∗∗*^ < 0.001.

## 3. Results

### 3.1. Patient Characteristics

We downloaded RNA-sequencing data and clinical prognostic information from TCGA database for 178 PAAD samples. As shown in Table 1, the clinical baseline table included age at diagnosis, sex, history of chronic pancreatitis, histological grade, pathological stage, overall survival, and disease-specific survival time.

### 3.2. mRNA Expression Level of COL10A1 in Pancreatic Cancer

**Figure 1:**
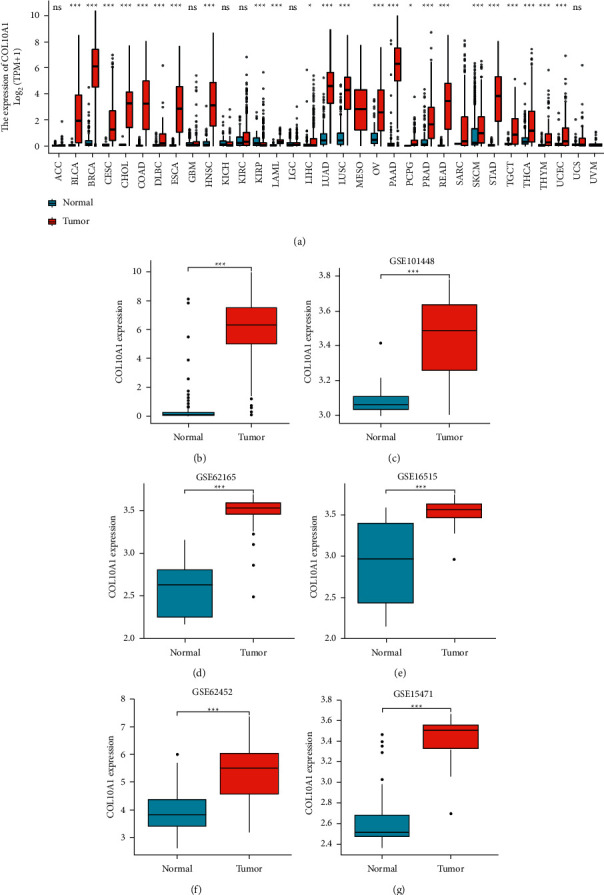
Expression level of COL10A1 in pan-cancer. (a) Expression levels of human COL10A1 in different cancer types from TCGA and GTEx data; (b)–(f) COL10A1 expression levels in PAAD versus normal tissues from GSE databases GSE15471, GSE101448, GSE62165, GSE16515, and GSE62452; (g) expression levels of COL10A1 in PAAD from TCGA and GTEx data. *p*^*∗*^ < 0.05; *p*^*∗∗*^ < 0.01; *p*^*∗∗∗*^ < 0.001.

**Figure 2:**
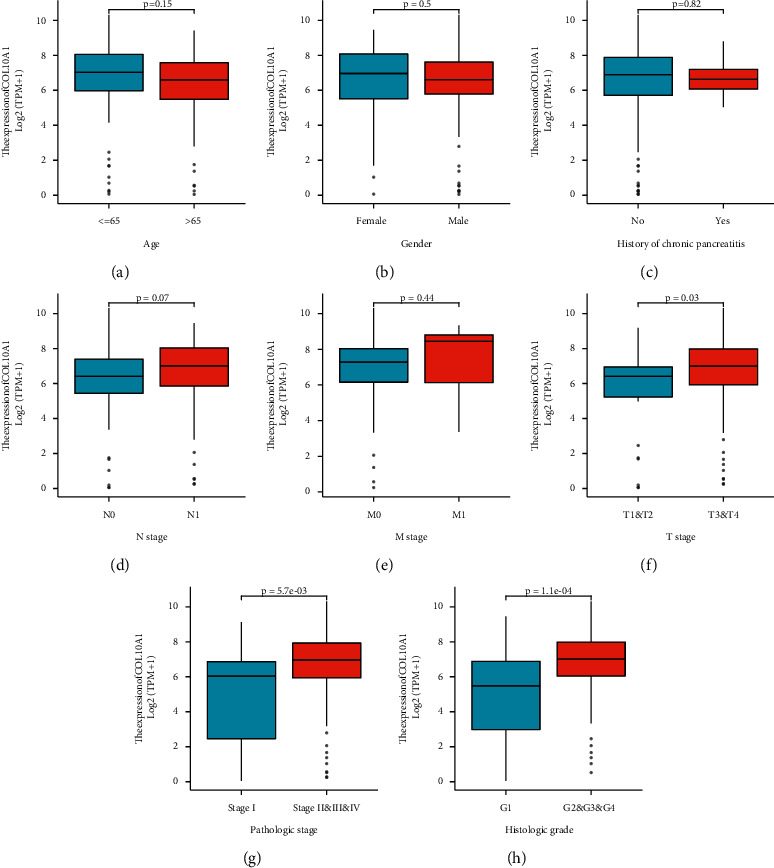
Correlation between COL10A1 and clinicopathological data of PAAD patients. (a)–(e) There were no significant differences between COL10A1 mRNA levels and age, gender, history of chronic pancreatitis, pathological stage N, and pathological stage M; (f)–(h) high COL10A1 expression was significantly correlated with higher T stage (T1 and T2 vs. T3 and T4), pathological stage (I vs. II–IV), and histological grade (G1 vs. G2 - G4).

The expression of COL10A1 in pan-cancer and adjacent normal tissues was first investigated. COL10A1 was significantly elevated in a variety of cancers, including pancreatic cancer, compared with normal tissues (Figure 1(a)). We then investigated RNA-sequencing data from TCGA and GTEx; COL10A1 expression was significantly higher in PAAD than in normal tissues (*p* = 1.9 *e* – 51; Figure 1(b)). When the gene expression levels of COL10A1 were analyzed using the GEPIA database, COL10A1 was significantly higher in PAAD than in normal tissues (*p* < 0.05; Supplement Figure 2). Using expression data mined from five GEO databases, we verified the differential expression of COL10A1 in PAAD (GSE15471: *p* = 2.9  *e* − 11; GSE101448: *p* = 9.3  *e* − 07; GSE62165: *p* = 6.6 *e* − 09; GSE16515: *p* = 1.1 *e* − 05; GSE62452 : *p* = 4.2  *e* – 13; Figures 1(c)–1(g)). Next, the correlation between COL10A1 and clinicopathological data of PAAD patients was analyzed. There were no significant differences between COL10A1 mRNA levels and age (*p* = 0.153; Figure 2(a)), gender (*p* = 0.5; Figure 2(b)), history of chronic pancreatitis (*p* = 0.87; Figure 2(c)), pathological stage *N* (*p* = 0.07; Figure 2(d)), and pathological stage *M* (*p* = 0.44; Figure 2(e)). COL10A1 expression was significantly higher in subgroups with higher stage/grade, such as *T* grade (T1-2 vs. T3-4), pathological stage (I vs. II–IV), and histological grade (G1 vs. G2-4) (T stage: *p* = 0.03; pathological stage: *p* = 5.7  *e* – 03; and histological grade: *p* = 1.1  *e* – 04; Figures 2(f)–2(h)).

### 3.3. The Prognostic Values of COL10A1 in Pancreatic Cancer

**Figure 3:**
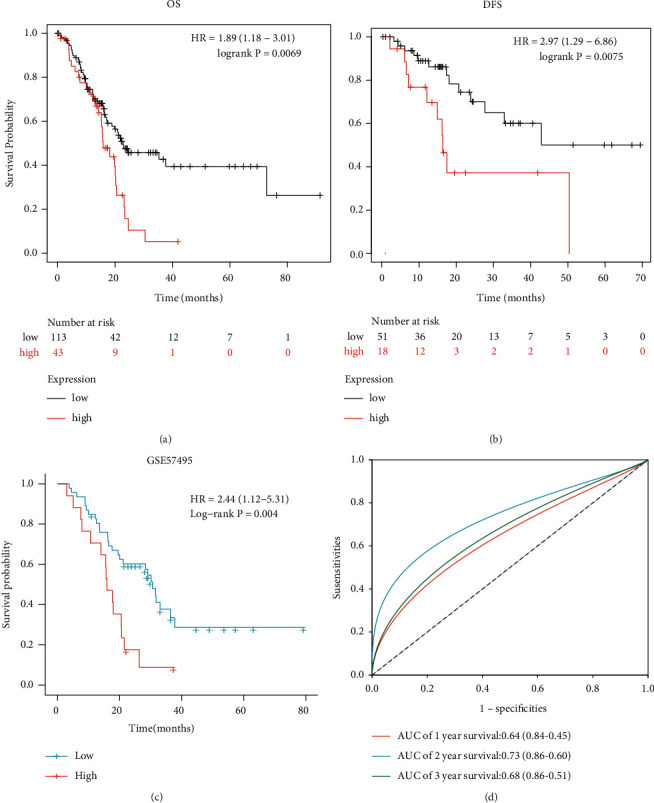
Survival analysis of COL10A1 in pancreatic cancer and normal tissues. (a) and (b) OS and DFS were analyzed by the Kaplan–Meier plotter; (c) Kaplan–Meier survival curves of COL10A1 in the GSE57495 dataset; (d) the ROC curves demonstrate the predictive power of the GSE57495 data.

We then performed a survival analysis of COL10A1 in PAAD. The Kaplan–Meier plotter shows that high COL10A1 expression was associated with poorer overall survival (OS) and disease-free survival (RFS) (OS : HR = 1.89, 95% CI = 1.18–3.01, *p* = 0.0069; DFS : HR = 2.97, 95% CI = 1.29–6.86, *p* = 0.0075; Figures 3(a) and 3(b)). The impact of COL10A1 expression on the prognosis of PAAD patients was verified by drawing the Kaplan–Meier survival curve using the GSE57495 dataset. The results showed that the high expression of COL10A1 was accompanied by a decrease in the overall survival of PAAD patients (OS : HR = 2.44, 95% CI = 1.12–5.31, *p* = 0.004; Figure 3(c)). The accuracy of the predictive power of survival curves in the GSE57495 dataset was demonstrated using ROC curves (AUC of 1-year survival: 0.64, 95% CI = 0.84–0.45; AUC of 2-year survival: 0.73, 95% CI = 0.86–0.60; and AUC of 3-year survival: 0.68, 95% CI = 0.86–0.51; Figure 3(d)). The univariate Cox regression analysis of COL10A1 in PAAD using clinical case data downloaded from TCGA showed that high COL10A1 expression, high stage (TNM), pathological grade (stage I vs. stages II–IV), and histological grade (G1 vs. G2–G4) were all negative predictors of OS and DSS in patients with PAAD (Table 2). This was confirmed by clinical data in TCGA.

### 3.4. Prediction of miRNA Upstream of COL10A1

**Figure 4:**
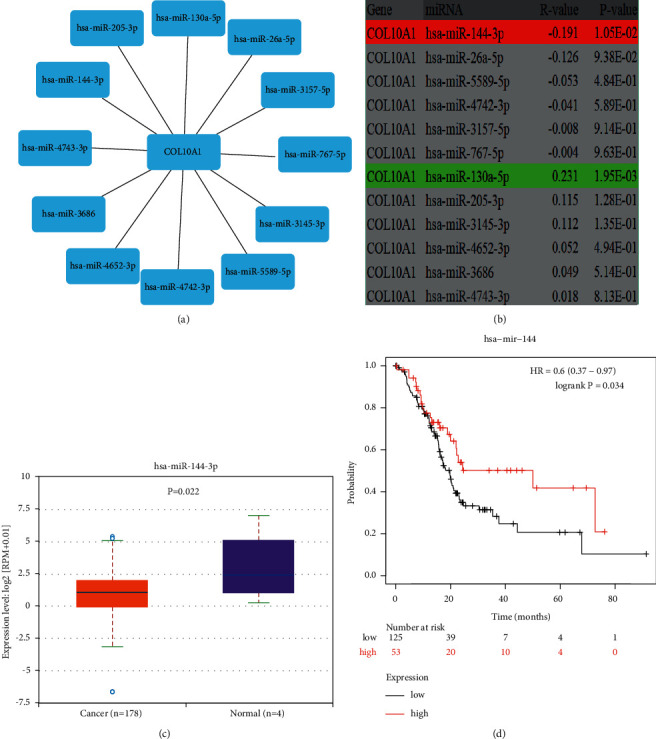
Construction of miR-144-3p/COL10A1 axis in PAAD. (a) Visualization of possible upstream miRNAs of COL10A1; (b) prediction of correlation between potential upstream miRNAs and COL10A1 expression in PAAD; (c) expression levels of miR-144-3p in PAAD; (d) impact of miR-144-3p expression on PAAD prognosis.

We utilized five prediction programs involving RNA22, miRmap, microT, miRDB, and TargetScan to predict the possible upstream microRNAs (miRNAs) of COL10A1. Setting the condition of being selected by two or more prediction programs simultaneously, 12 miRNAs were finally obtained (Figure 4(a)). According to the competing endogenous RNA (ceRNA) hypothesis, miRNAs and COL10A1 should be negatively correlated. Therefore, a correlation analysis was performed for the predicted 12 miRNAs. In pancreatic cancer, COL10A1 was significantly negatively correlated with hsa-miR-144-3p only (Figure 4(b)). It indicates that miR-144-3p may be the upstream miRNA of COL10A1. Next, the expression of miR-144-3p in PAAD was analyzed, and miR-144-3p was significantly downregulated in PAAD (Figure 4(c)). The Kaplan–Meier plotter showed that high expression of hsa-miR-144-3p was associated with better overall survival (Figure 4(d)). In combination with the above results, hsa-miR-144-3p may be the upstream miRNA for COL10A1.

### 3.5. Upstream lncRNA Prediction of miR-144-3p

**Figure 5:**
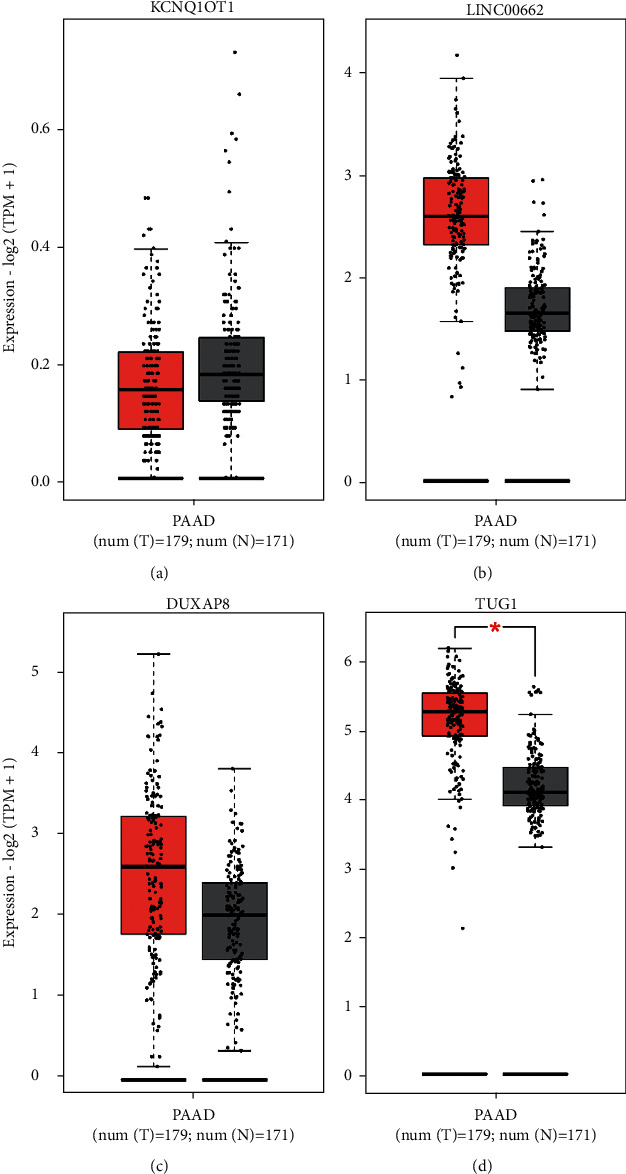
Online database GEPIA validated the upstream lncRNAs of the miR-144-3p/COL10A1 axis using “TCGA and GTEx normal” data. The results show KCNQ1OT1 (a), LINC00662 (b), DUXAP8 (c), and TUG1 (d).

**Figure 6:**
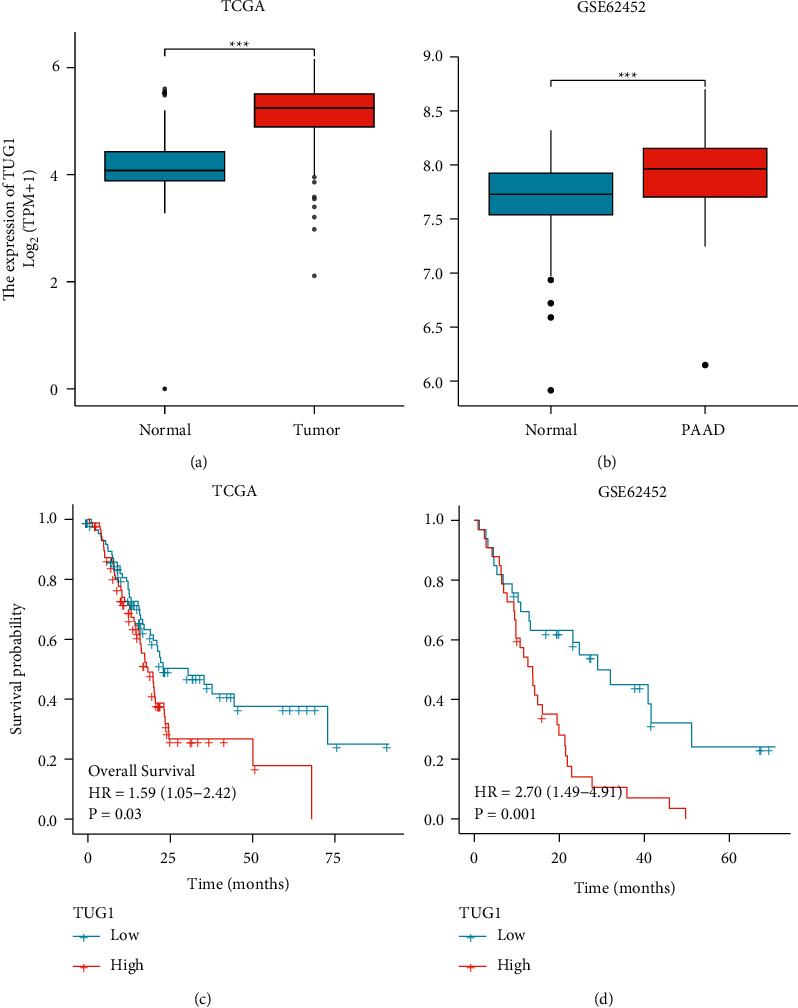
Validation of TUG1 as an upstream lncRNA of miR-144-3p/COL10A1 axis. (a) Expression of TUG1 in PAAD, based on TCGA data; (b) expression of TUG1 in PAAD, based on GSE62452 dataset; (c) and (d) the impact of TUG1 on the overall survival of PAAD was studied using TCGA database and verified using the GSE62452 dataset. *p*^*∗*^ < 0.05; *p*^*∗∗*^ < 0.01; *p*^*∗∗∗*^ < 0.001.

**Figure 7:**
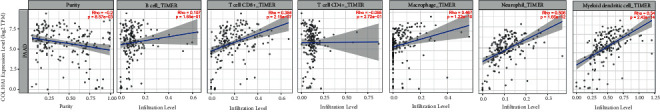
Correlation between COL10A1 expression and immune cell markers in PAAD.

In recent years, long noncoding RNAs (lncRNAs) are major regulators of gene expression, and as such, they can regulate important cellular signaling pathways in cancer. A total of 96 lncRNAs were predicted upstream of the miR-144-3p/COL10A1 axis using StarBase. A total of 96 lncRNAs were visualized using the Cellscape software (Supplement Figure 3). According to the competitive endogenous RNA hypothesis, microRNAs are known to cause gene silencing by binding messenger RNA (mRNA), while lncRNAs can increase gene expression by competitively binding microRNAs. Therefore, there should a positive correlation between lncRNAs and mRNAs. Only four lncRNAs are consistent with the hypothesis of competing for endogenous RNAs (Table 3). The expression of these four lncRNAs in PAAD was then analyzed using the GEPIA online database. As shown in Figures 5(a)–6(d), only TUG1 among these four lncRNAs was significantly upregulated in pancreatic cancer compared with “TCGA and GTEx normal” data (*p* < 0.05). And the high expression of TUG1 in PAAD was verified using TUG1 expression data from TCGA database and the GEO dataset GSE62452 (*p* < 0.001) (Figures 6(a)–7(b)). Clinical information was extracted from the database TCGA and the dataset GSE62452, respectively, and Kaplan–Meier (KM) survival curves were used to compare survival differences, and the results showed that pancreatic cancer patients with TUG1 upregulation exhibited poor OS (Figures 6(c)–7(d)). Taken together, TUG1 may be the most promising upstream lncRNA of the miR-144-3p. In addition, we also performed a prognostic analysis of TUG1 using GSE57495, which showed a worse prognosis for high TUG1 expression, but the *p* value was not significant (HR = 1.59 (0.87–2.92), *p* = 0.139). This may be due to the small sample size in GSE57495.

### 3.6. Correlation between COL10A1 and Immune Infiltration

Tumor-infiltrating immune cells (TIICs) mainly include CD8+ T cells, CD4+ T cells, B cells, neutrophils, macrophages, and dendritic cells, which account for a large proportion of TME [30]. The scatter plot shows the correlation between COL10A1 expression levels and the abundance of six tumor-infiltrating immune subgroups in PAAD. COL10A1 expression levels were positively correlated with CD8+T cells (*r* = 0.384, *p* = 2.15 *e* − 07), macrophages (*r* = 0.467, *p* = 1.22 *e* − 10), neutrophils (*r* = 0.506, *p* = 1.66 *e* − 12), and dendritic cells (*r* = 0.54, *p* = 2.14 *e* − 14), and negatively correlated with tumor purity in pancreatic cancer (*r* = −0.2, *p* = 8.57 *e* − 03) but not with CD4+ T cells and B cells (Figure 7).

Immune cell markers are considered to be the corresponding symbols of different immune cells [31]. To further investigate the correlation between COL10A1 expression levels and TIICs, we investigated the relationship between COL10A1 expression levels and infiltration levels in six tumor-infiltrating immune subpopulations using immune cell markers corresponding to different immune cells. As shown in Table 4, after adjusting for tumor purity, COL10A1 expression levels were statistically related to 38 of the 55 pancreatic cancer immune cell markers.

### 3.7. Association between COL10A1 and Immune Checkpoints and Prognostic Markers in PAAD

**Figure 8:**
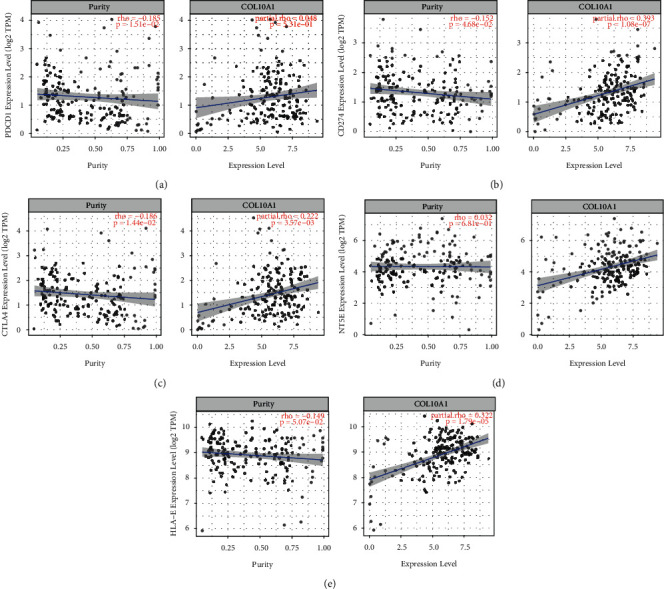
COL10A1 expression correlates with immune checkpoints and other tumor markers in PAAD. The results show PD-1 (a), PD-L1 (b), CTLA-4 (c), CD73 (d), and HLA-E (e).

Immune checkpoint inhibitors (ICIs) such as anti-programmed cell death 1 (PDCD1/PD1), anti-programmed death-ligand 1 (PD-L1/CD274), and anti-cytotoxic T-lymphocyte-associated protein 4 (CTLA-4) are approved for immunotherapy [32]. PD1/PD-L1 and CTLA-4 are involved in the immune escape from tumors and are immune checkpoints for immune escape, when a tumor antagonizes, blocks, and suppresses the body's immune response through its structural and nonstructural products. We analyzed the correlation between COL10A1 expression levels and immune checkpoint expression in PAAD. After adjusting for purity, COL10A1 expression was positively correlated with PD-L1 and CTLA-4 in PAAD. However, COL10A1 had no relationship with PD1 expression (Figures 8(a)–8(c)). We also correlated COL10A1 with other prognostic markers in PAAD using TIMER. CD73 promotes immune escape in pancreatic cancer [33]. In PAAD, CD73 was significantly positively correlated with COL10A1 (Figure 8(d)). Human leukocyte antigen (HLA)-E inhibits the antitumor effect of NK cells [34]. HLA-E expression is elevated in PDAC and contributes to poor prognosis [35]. Also using TIMER, COL10A1 was significantly positively correlated with HLA-E (Figure 8(e)). The above results suggest that COL10A1 causes poor prognosis in PAAD, probably because of involvement in immune escape.

## 4. Discussion

PAAD is one of the most lethal tumors due to its difficulty in early diagnosis [1]. Elucidation of the molecular mechanisms underlying the pathogenesis of PAAD can help in the noninvasive and early diagnosis of cancer, the establishment of prognostic panels, and the development of effective targeted therapies. Increasing evidence suggests that COL10A1 is associated with the malignant progression of multiple cancers [9–11]. However, studies on COL10A1 in PAAD are still insufficient and further research is needed.

We first analyzed the expression pattern and prognosis of COL10A1 in pancreatic cancer. Combined with validation from multiple databases, the expression of COL10A1 in pancreatic cancer tissues was significantly higher than that in normal tissues. Correlation with clinicopathological features indicated that COL10A1 expression was significantly higher in the high stage/grading subgroup. Subsequently, survival curves showed that high COL10A1 expression predicted shorter survival in PAAD patients. The univariate analysis showed that COL10A1, histological grade, and pathological stage were important predictors of survival in pancreatic cancer patients. In conclusion, COL10A1 may be involved in the malignant transformation process of PAAD and may be a biomarker of PAAD.

Previous studies have shown that miRNAs can cause gene silencing by binding to mRNAs, while ceRNAs can regulate tumorigenesis, invasion, metastasis, and drug resistance by competitively binding miRNAs [36]. We used multiple online prediction tools to predict the possible upstream miRNAs of COL10A1 and finally obtained 12 miRNAs. According to the competing endogenous RNA (ceRNA) hypothesis, there should be a negative correlation between microRNAs and COL10A1. Therefore, expression correlation analysis was performed for the predicted 12 miRNAs, and only miR-144-3p was negatively correlated with COL10A1 in PAAD. Next, expression analysis and prognostic analysis were performed for miR-144-3p in PAAD. hsa-miR-144-3p was significantly downregulated in PAAD, and higher levels of hsa-miR-144-3p were associated with better overall survival. This suggests that miR-144-3p is a protective factor in PAAD, and according to the competing endogenous RNA (ceRNA) hypothesis, miR-144-3p may be the most promising upstream miRNA for COL10A1. In addition, several previous studies demonstrated the protective effect of miR-144-3p against tumors, such as lung cancer [37], liver cancer [38], colorectal cancer [39], renal carcinoma [40], and breast cancer [41]. miR-144-3p expression is downregulated in pancreatic cancer tissues and cell lines [42, 43]. The transfection of pancreatic cancer cell lines (PANC-1) using miR-144-3p resulted in diminished colony formation of pancreatic cancer cells and a significant decrease in cell invasion and migration (*p* < 0.01) [42]. Li et al found that miR-144-3p could arrest pancreatic cancer cells in the S phase of the cell cycle by activating the mitogen-activated protein kinase pathway, and its inhibitory effect on pancreatic cancer cell proliferation could be reversed using miR-144-3p inhibitors [43].

The ceRNA hypothesis reveals a novel mechanism of lncRNA/miRNA/mRNA interactions [44]. This study predicted 96 lncRNAs upstream of miR-144-3p. Based on the ceRNA hypothesis, Tug1 was identified as the most likely upstream lncRNA to be upregulated. In addition, several studies have shown the relevance of TUG1 to pancreatic cancer. For example, Qin and Zhao demonstrated that lncRNA TUG1 can promote the malignant progression of pancreatic cancer via the EMT pathway [45]. Yang et al. demonstrated that TUG1 affects tumor invasion and gemcitabine resistance in pancreatic cancer [46]. Liang et al. demonstrated that TUG1 may be a potential target for the treatment of PAAD [47]. Taken together, TUG1/miR-144-3p/COL10A1axis was identified as potential regulatory pathways in PAAD.

Tumor immune cells are part of the tumor microenvironment and, in addition to their antitumor effects, also lead to immune escape from tumors, which in turn promotes tumor development [14, 15]. Infiltration of tumor immune cells affects the efficacy and prognosis of chemotherapy, radiotherapy, or immunotherapy in tumor patients [48–50]. Correlation analysis showed that the expression level of COL10A1 was negatively correlated with the tumor purity of PAAD, indicating its relative enrichment in the tumor microenvironment. COL10A1 expression was closely associated with CD8+ T cells and their markers. CD8+ T cells are effector cells for immunotherapy [51]. Activated CD8+ T cells can kill tumor cells by perforating proteases of the Fas/Fas ligand pathway [52]. Further analysis showed that COL10A1 expression levels were associated with M1 macrophages, M2 macrophages, tumor-associated markers of macrophage (TAM), and T-regulatory (Treg) cells. In early cancer progression, TAM tends to favor the M1 phenotype and promotes antitumor activity. In contrast, as the tumor progresses, TAM has the opposite effect; TAM prefers the M2 phenotype and promotes tumor immunoregulation, thus promoting tumor invasion and malignant progression [53]. The imbalance in the ratio of M1/M2 macrophage subpopulations may be related to the procarcinogenic effect of COL10A1. Treg cells have immunosuppressive effects and can reduce the activity of effector cells. In addition, we found that COL10A1 was significantly associated with CD11c + DC, which is a key target of Treg cells and is involved in immunosuppression in the tumor microenvironment [54]. Taken together, tumor immune infiltration may partially explain the oncogenic effect of COL10A1-mediated pancreatic cancer.

Tumor immunotherapy, such as immune checkpoint inhibitors based on monoclonal antibodies, clears tumors by rebooting the organism's normal antitumor immune response [55]. PD-1/PD-L1 and CTLA-4 are immune checkpoints for immunotherapy [56]. We investigated the correlation between COL10A1 and immune checkpoints. The results showed that COL10A1 was significantly correlated with PD-L1 and CTLA-4 in pancreatic cancer, suggesting that COL10A1 may be associated with immune escape. COL10A1 may serve as a new target for immunotherapy.

Our study also has shortcomings. The main limitation of this study is that the results were based on bioinformatics analysis and the data were mainly obtained from public databases, and there were heterogeneity of the data and platform differences among the different databases [57]. We finally validated the promoting role of COL10A1 in cancer development by validating it against several different databases. However, this result needs to be verified by further laboratory experiments.

## 5. Conclusion

Our study suggests that COL10A1 overexpression is associated with poor prognosis in PAAD and can be used as a biomarker for PAAD. We depicted a TUG1/miR-144-3p/COL10A1 axis to investigate the regulatory mechanism of COL10A1 in pancreatic cancer progression. In addition, we performed a functional analysis of COL10A1. COL10A1 is involved in immune infiltration in PAAD, is associated with immunosuppression, and negatively affects the effect of immunotherapy. However, these findings need to be validated by further laboratory experiments.

## Figures and Tables

**Table 1:** Clinical characteristics of PAAD patients in TCGA database.

Characteristics	Levels	Overall
*n*		178
T stage, *n* (%)	T1	7 (4%)
	T2	24 (13.6%)
	T3	142 (80.7%)
	T4	3 (1.7%)

N stage, *n* (%)	N0	50 (28.9%)
	N1	123 (71.1%)

M stage, *n* (%)	M0	79 (94%)
	M1	5 (6%)

Pathological stage, *n* (%)	Stage I	21 (12%)
	Stage II	146 (83.4%)
	Stage III	3 (1.7%)
	Stage IV	5 (2.9%)

Gender, *n* (%)	Female	80 (44.9%)
	Male	98 (55.1%)

Age, *n* (%)	≤65	93 (52.2%)
	>65	85 (47.8%)

Histological grade, *n* (%)	G1	31 (17.6%)
	G2	95 (54%)
	G3	48 (27.3%)
	G4	2 (1.1%)

OS event, *n* (%)	Alive	86 (48.3%)
	Dead	92 (51.7%)

DSS event, *n* (%)	Alive	100 (58.1%)
	Dead	72 (41.9%)

T stage, primary tumor size; N stage, regional lymph nodes; *M* stage, distant metastasis; OS, overall survival; DSS, disease specific survival.

**Table 2:** Correlation with clinicopathological characteristics of PAAD patients analyzed by the Cox regression analysis.

Characteristics	Total (*N*)	Univariate analysis (OS)		Univariate analysis (DSS)	
		Hazard ratio (95% CI)	*p* value	Hazard ratio (95% CI)	*p* value
Age (>65vs. ≤ 65)	178	0.775 (0.513–1.171)	0.227	0.937 (0.588–1.493)	0.784
Gender (male vs. female)	178	0.809 (0.537–1.219)	0.311	0.751 (0.473–1.194)	0.227
T stage (T1and T2 vs. T3 and T4)	176	2.023 (1.072–3.816)	0.030	1.207 (1.066–1.367)	0.003
N stage (N0 vs. N1)	173	2.154 (1.282–3.618)	0.004	2.746 (1.473–5.121)	0.001
M stage (M1 vs. M0)	84	1.323 (0.317–5.525)	0.701	1.116 (0.265–4.708)	0.881
COL10A1	178	1.179 (1.059–1.313)	0.003	1.207 (1.066–1.367)	0.003
Histological grade (G1 vs. G2 and G3 and G4)	176	2.164 (1.139–4.110)	0.018	2.101 (1.032–4.278)	0.041
Pathological stage (stage I vs. stages II and III and IV)	175	2.291 (1.051–4.997)	0.037	3.249 (1.175–8.979)	0.023
History of chronic pancreatitis (yes vs. no)	141	1.177 (0.562–2.464)	0.666	0.888 (0.354–2.232)	0.801

T stage, primary tumor size; N stage, regional lymph nodes; *M* stage, distant metastasis.

**Table 3:** Correlation between 4 lncRNAs and miR-144-3p and between 4 lncRNAs and COL10A1.

	miR-144-3p		COL10A1	
	COR	*p* value	COR	*p* value
KCNQ1OT1	−0.231	1.88 *E* − -03	0.15	0.047
LINC00662	−0.158	3.56 *E* − 02	0.27	3.10 *E* − 04
DUXAP8	−0.163	2.94 *E* − 02	0.43	2.5 *E* − 09
TUG1	−0.194	9.37 *E* − 03	0.38	1.3 *E* − 07

lncRNAs negatively associated with miR-144-3p expression and positively associated with COL10A1 expression include KCNQ1OT1, LINC00662, DUXAP8, and TUG1. Cor: *R* value of Pearson's correlation; bold values indicate *p* < 0:05.

**Table 4:** Correlation between COL10A1 and markers of immune cells in TIMER and GEPIA.

Cell type	Gene marker	PAAD			
		None		Purity	
		Cor	*p*	Cor	*p*
B cell	CD19	0.177	0.0176	0.126	0.096
	CD20 (krtx20)	0.056	0.4	0.4	0.055
	CD38	0.3	*∗∗∗*	0.26	*∗∗*
CD8+T cell	CD8A	0.207	*∗*	0.152	0.0474
	CD8B	0.187	0.0123	0.126	0.1
Tfh	CXCR5	0.059	0.444	0.119	0.114
	LCOS	0.305	*∗∗∗*	0.266	*∗∗∗*
	BCL-6	0.321	*∗∗∗*	0.3	*∗∗∗*
Th1	IL-12RB2	0.023	0.756	-0.007	0.931
	WSX-1 (IL-27RA)	0.247	*∗∗*	0.204	*∗*
	T-BET (TBX21)	0.102	0.175	0.064	0.4
	STAT1	0.476	*∗∗∗*	0.443	*∗∗∗*
	IFN-*γ* (IFNG)	0.258	*∗∗*	0.225	*∗*
	TNF-*α* (TNF)	0.279	*∗∗*	0.251	*∗∗*
Th2	CCR3	0.368	*∗∗∗*	0.308	*∗∗∗*
	STAT6	0.178	*∗*	0.195	*∗*
	GATA-3	0.289	*∗∗*	0.307	*∗∗∗*
	STAT5A	0.274	*∗∗*	0.236	*∗*
Th9	TGF-BR2	0.43	*∗∗∗*	0.392	*∗∗∗*
	IRF4	0.257	*∗∗*	0.199	*∗*
	PU.1 (SPI1)	0.347	*∗∗∗*	0.313	*∗∗∗*
Th17	IL-21R	0.454	*∗∗∗*	0.494	*∗∗∗*
	IL-23R	0.018	0.8	0.094	0.2
	STAT3	0.431	*∗∗∗*	0.494	*∗∗∗*
Th22	CCR10	-0.035	0.639	-0.041	0.597
	ARH	0.454	*∗∗∗*	0.407	*∗∗∗*
Treg	FOXP3	0.445	*∗∗∗*	0.411	*∗∗∗*
	CCR8	0.527	*∗∗∗*	0.498	*∗∗∗*
	CD25 (IL-2RA)	0.445	*∗∗∗*	0.402	*∗∗∗*
T-cell exhaustion	PD-1 (PDCD1)	0.116	0.122	0.048	0.531
	CTLA4	0.273	*∗∗∗*	0.222	*∗*
	TIM-3 (HAVCR2)	0.553	*∗∗∗*	0.514	*∗∗∗*
Macrophage	CD68	0.541	*∗∗∗*	0.499	*∗∗∗*
	CD11b (ITGAM)	0.494	*∗∗∗*	0.437	*∗∗∗*
M1	NOS2	0.278	*∗∗∗*	0.282	*∗∗∗*
	IRF5	0.213	*∗*	0.222	*∗*
M2	ARG1	-0.067	0.375	0.039	0.612
	MRC1	0.391	*∗∗∗*	0.395	*∗∗∗*
	MS4A4A	0.471	*∗∗∗*	0.42	*∗∗∗*
TAM	HLA-G	0.111	0.137	0.063	0.413
	CD80	0.535	*∗∗∗*	0.514	*∗∗∗*
	CD86	0.525	*∗∗∗*	0.489	*∗∗∗*
	CCR5	0.338	*∗∗∗*	0.278	*∗*
Monocyte	CD14	0.363	*∗∗∗*	0.307	*∗∗∗*
	CD16 (FCGR3B)	0.384	*∗∗∗*	0.326	*∗∗∗*
	CD115 (CSF1R)	0.411	*∗∗∗*	0.349	*∗∗∗*
NK	XCL1	0.127	0.0899	0.098	0.2
	KIR3DL1	0.093	0.216	0.07	0.363
	CD7	0.115	0.125	0.061	0.425
Neutrophil	CD15 (FUT4)	0.24	*∗*	0.183	0.164
	MPO	0.318	*∗∗∗*	0.327	*∗∗*
	CD11b (ITGAM)	0.494	*∗∗∗*	0.437	*∗∗∗*
DC	CD1C (BDCA-1)	0.187	0.0124	0.148	0.0528
	CD141	0.232	*∗*	0.228	*∗*
	CD11c (ITGAX)	0.452	*∗∗∗*	0.403	*∗∗∗*

Tfh, follicular helper T cell; Th, *T* helper cell; Treg, regulatory T cell; TAM, tumor-associated macrophage; none, correlation without adjustment; purity, correlation adjusted by purity; Cor, *R* value of Spearman's correlation. ^*∗*^*p* < 0.05; ^*∗∗*^*p* < 0.01; ^*∗∗∗*^*p* < 0.001.

## Data Availability

The open dataset is available at the following URL : GSE15471 (https://www.ncbi.nlm.nih.gov/geo/query/acc.cgi?acc=GSE15471), GSE101448 (https://www.ncbi.nlm.nih.gov/geo/query/acc.cgi?acc=GSE101448), GSE62165 (https://www.ncbi.nlm.nih.gov/geo/query/acc.cgi?acc=GSE62165), GSE16515 (https://www.ncbi.nlm.nih.gov/geo/query/acc.cgi?acc=GSE16515), GSE57495 (https://www.ncbi.nlm.nih.gov/geo/query/acc.cgi?acc=GSE57495), GSE62452 (https://www.ncbi.nlm.nih.gov/geo/query/acc.cgi?acc=GSE62452), TCGA (https://genomecancer.ucsc.edu/), and PANCAN, *N* = 19131, *G* = 60499 (https://xenabrowser.net/). The datasets used in this study are available from the corresponding author upon reasonable request.

## Abbreviations:

COL10A1: Collagen type X alpha 1
TME: Tumor microenvironment
PAAD: Pancreatic cancer
TAM: Tumor-associated markers of macrophage
Treg: T-regulatory
HR: Hazard ratio
CI: Confidence interval.

## Supplementary Materials

Supplementary Materials Supplement Figure 1: Functional Enrichment Analysis of Genes Coexpressed with COL10A1. Supplement Figure 2: Expression levels of COL10A1 in PAAD versus normal tissues from the GEPIA database. Supplement Figure 3: Ninety-six possible upstream lncRNAs predicted by StarBase. Supplementary Table 1: Intersection of the UALCAN database and the GEPIA database for coexpressed genes. Supplementary Table 2: Functional Enrichment Analysis of Genes Coexpressed with COL10A1.
