# Supplementary material for: Bioinformatics-Based Analysis: Noncoding RNA-Mediated COL10A1 Is Associated with Poor Prognosis and Immune Cell Infiltration in Pancreatic Cancer
Source: J Healthc Eng. 2022 Sep 5;2022:7904982. doi: 10.1155/2022/7904982 (PMC9467764; doi:10.1155/2022/7904982)

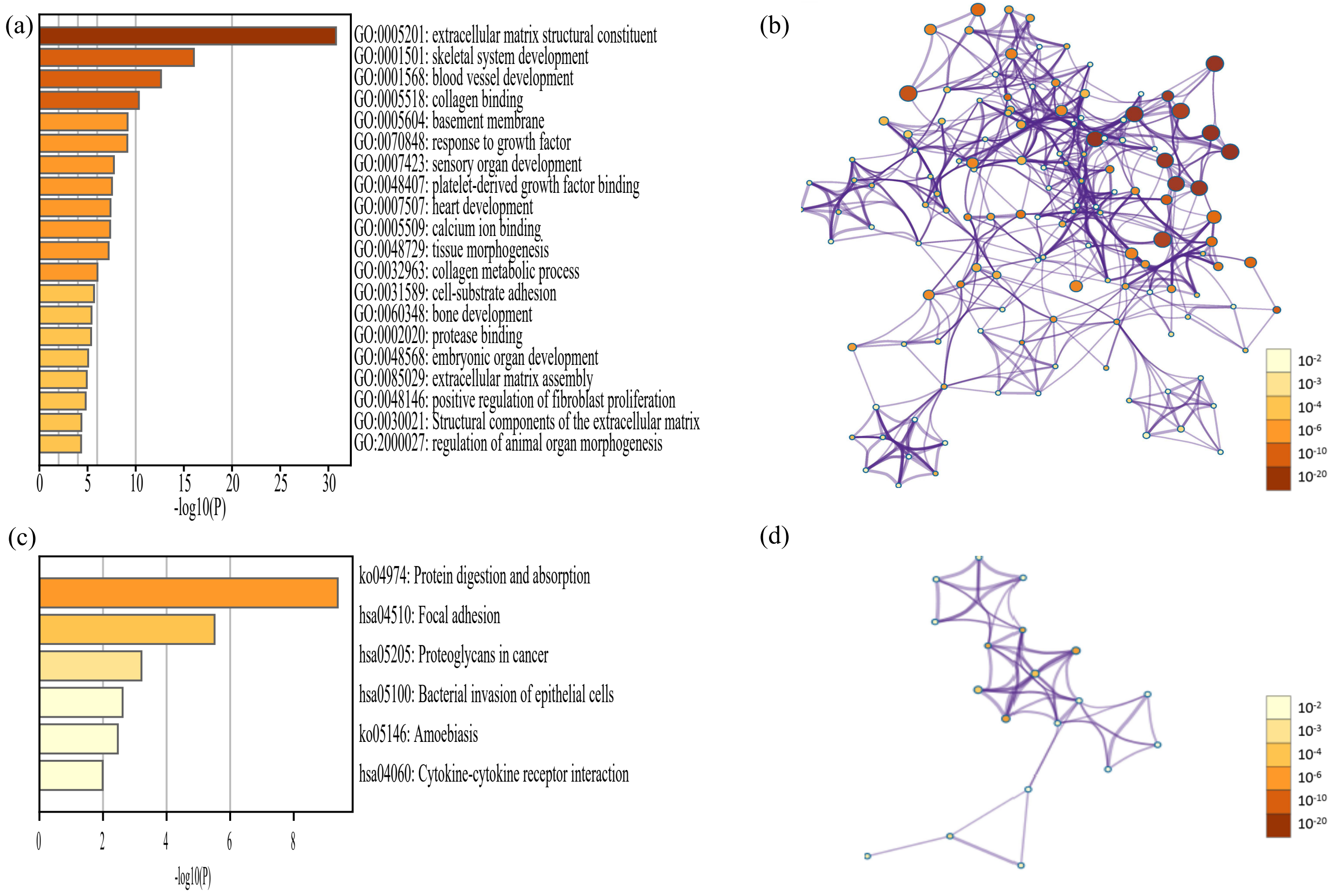

(e)

Color	MCODE	GO	Description	Log10(P)
(red)	MCODE_1	M3005	NABA collagens Collagen	-28.5
(red)	MCODE_1	R-HSA-8948216	chain trimerization	-28.5
(red)	MCODE_1	M198	PIO syndecan 1 pathway	-28.3
(blue)	MCODE_2	R-HSA-8957275	Post-translational protein phosphorylation	-17.0
(blue)	MCODE_2	R-HSA-381426	Regulation of Insulin-like Growth Factor (IGF) transport and uptake by Insulin-like Growth Factor Binding Proteins (IGFBPs)	-16.5
(blue)	MCODE_2	R-HSA-2129379	Molecules associated with elastic fibres	-7.1

(f)

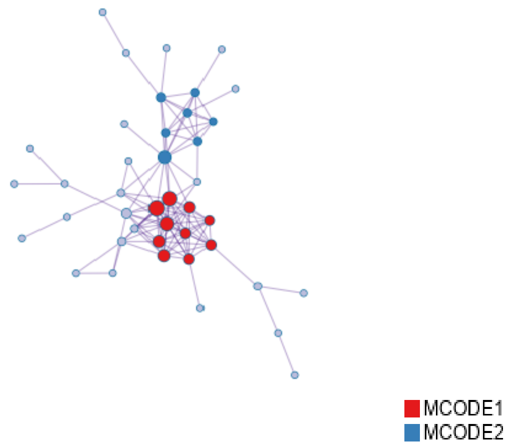

Supplement: Supplementary Materials — Supplement Figure 1: Functional Enrichment Analysis of Genes Coexpressed with COL10A1. Supplement Figure 2: Expression levels of COL10A1 in PAAD versus normal tissues from the GEPIA database. Supplement Figure 3: Ninety-six possible upstream lncRNAs predicted by StarBase. Supplementary Table 1: Intersection of the UALCAN database and the GEPIA database for coexpressed genes. Supplementary Table 2: Functional Enrichment Analysis of Genes Coexpressed with COL10A1. [file 7904982.f1.zip › 7904982.f1/Supplementary Figure. 1.pdf]
