# Supplementary material for: Bioinformatics-Based Analysis: Noncoding RNA-Mediated COL10A1 Is Associated with Poor Prognosis and Immune Cell Infiltration in Pancreatic Cancer
Source: J Healthc Eng. 2022 Sep 5;2022:7904982. doi: 10.1155/2022/7904982 (PMC9467764; doi:10.1155/2022/7904982)
Supplement: Supplementary Materials — Supplement Figure 1: Functional Enrichment Analysis of Genes Coexpressed with COL10A1. Supplement Figure 2: Expression levels of COL10A1 in PAAD versus normal tissues from the GEPIA database. Supplement Figure 3: Ninety-six possible upstream lncRNAs predicted by StarBase. Supplementary Table 1: Intersection of the UALCAN database and the GEPIA database for coexpressed genes. Supplementary Table 2: Functional Enrichment Analysis of Genes Coexpressed with COL10A1. [file 7904982.f1.zip › 7904982.f1/Supplementary file (1).docx]

**Functional Enrichment Analysis of Genes Co-expressed with COL10A1**

Enrichment analysis will allow us to better understand the function of COL10A1 in PAAD. Genes co-expressed with COL10A1 were screened in the UALCAN and GEPIA databases, respectively, and there were 87 identical co-expressed genes in both data (**Supplementary table1**). GO and KEGG functional analysis of COL10A1 and its co-expressed genes were performed using Metascape. The top 20 GO enrichment items were shown in **Supplement Figure.1 (a) and (b); Supplementary table.2**. The GO functional enrichment of these co-expressed genes in the biological process categories includes skeletal system development (GO:0001501), vascular development (GO:0001568), response to growth factors (GO:0070848), sensory organ development (GO:0007423), cardiac development (GO:0007507), tissue morphogenesis (GO: 0048729), collagen metabolic processes (GO:0032963), cell-matrix adhesion (GO:0031589), bone development (GO:0060348), embryonic organ development (GO: 0048568), extracellular matrix assembly (GO:0085029), positive regulation of fibroblast proliferation (GO:0048146 ), and regulation of animal organ morphogenesis (GO:2000027). In the Cellular Components category, it included basement membrane (GO:0005604). Finally, the molecular functional group includes the following: extracellular matrix structural components (GO:0005201), collagen binding (GO: 0005518), platelet-derived growth factor binding (GO:0048407), calcium-binding (GO:0005509), protease binding (GO:0002020), and structural components of fibroblast proliferation (GO:0030021). The top 6 KEGG pathways were as follows: protein digestion and uptake, focal adhesion, proteoglycans in cancer, bacterial invasion of epithelial cells, amebiasis, and cytokine-cytokine receptor interactions (**Supplement Figure.1(c) and (d); Supplementary table 2**). In addition, to better investigate the role of COL10A1 in PAAD, we then used the protein-protein interaction network structure analysis that comes with the Metascape online tool for enrichment analysis, As shown in **Supplement Figure.1(e) and (f)**. A total of two important MCODE components were obtained and analyzed for pathway and process enrichment, respectively. The main relevant biological functions are NABA Collagens, collagen chain trimerization, PID syndecan 1 pathway, post-translational protein phosphorylation, insulin-like growth factor binding proteins for an insulin-like growth factor (IGF) transport and uptake regulation, and molecules related to elastic fibers.

Supplement Figure.1


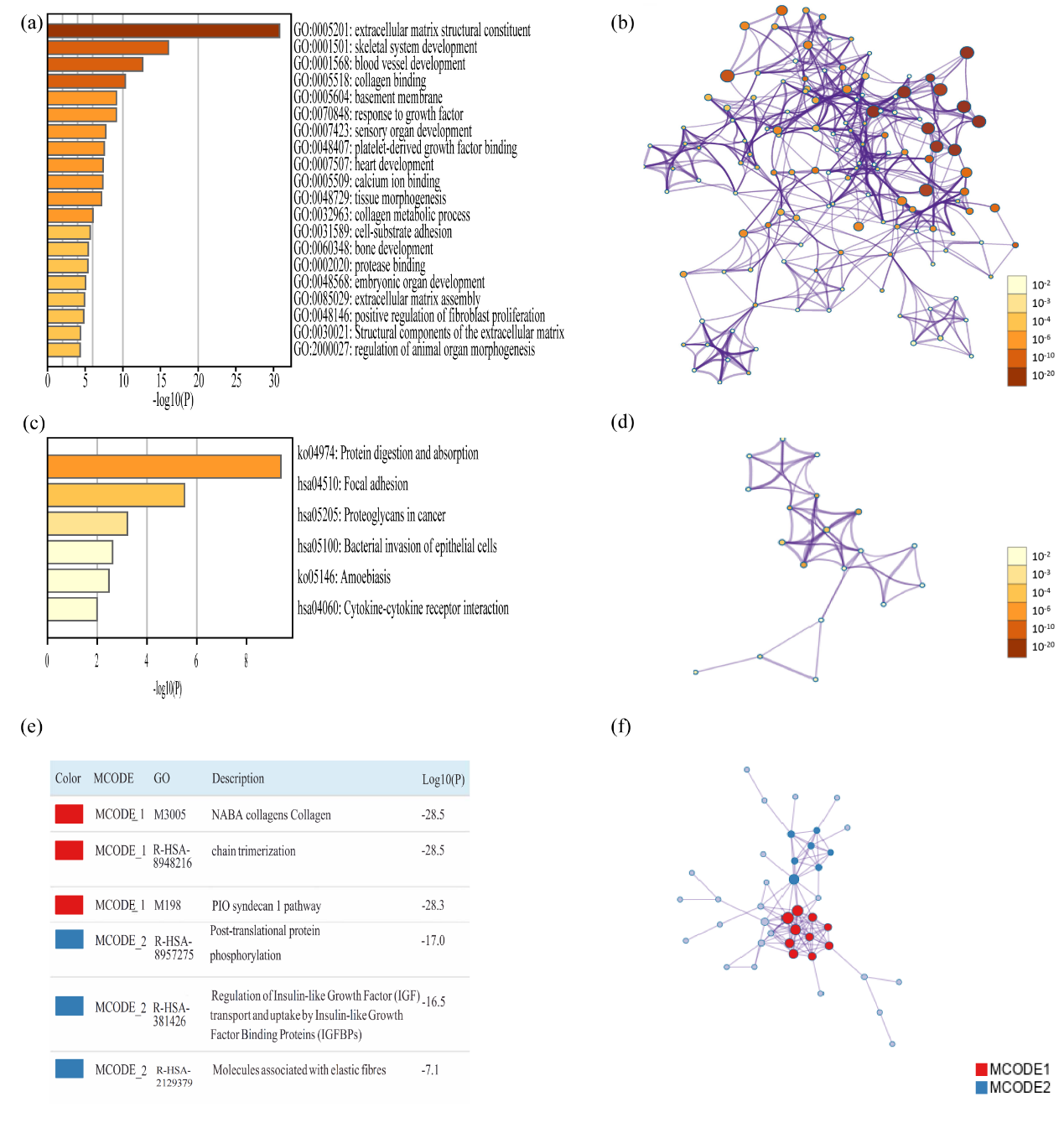


Supplement Figure.1: Functional enrichment analysis of COL10A1 and its co-expressed genes. **(a)-(b)** The top 20 GO enrichment terms and the most statistically significant term were selected for visual network mapping. **(c)-(d)** Top 6 KEGG pathways and select statistically significant terms as visual network mapping. **(e)-(f)** Protein-protein interaction network structure analysis and MCODE components identified in the gene list.

Supplement Figure.2


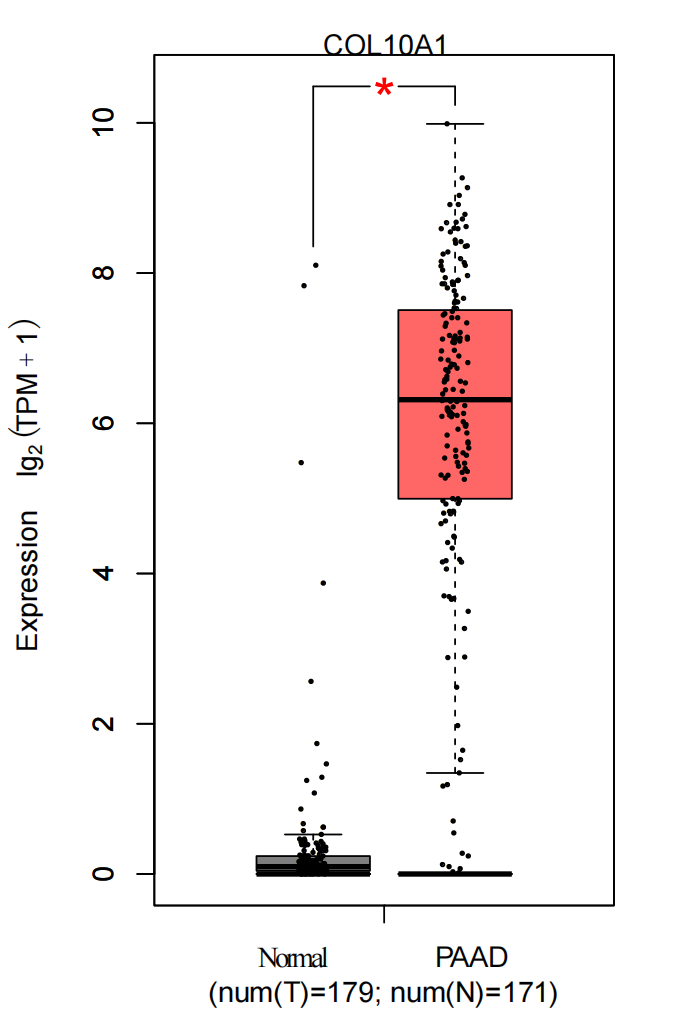


Supplement Figure.2: Expression levels of COL10A1 in PAAD versus normal tissues from GEPIA database. Abbreviation: PAAD, pancreatic cancer; COL10A1, Collagen type X alpha 1

Supplement Figure.3


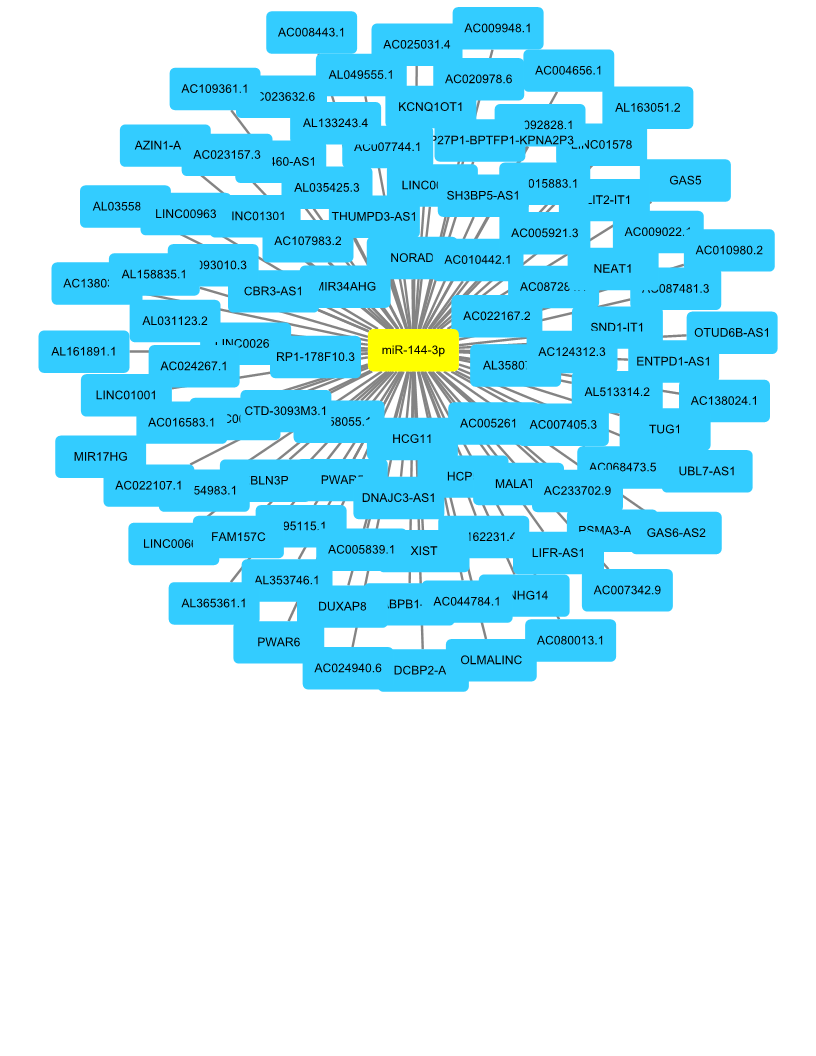
Supplement Figure.3: The 96 possible upstream lncRNAs predicted by Starbase.

| Supplementary table 1: Intersection of the UALCAN database and the GEPIA database for co-expressed genes. | | | | | | | |
| --- | --- | --- | --- | --- | --- | --- | --- |
| UALCAN AND GEPIA | THBS2 | VCAN | ST6GAL2 | ZFHX4 | FKBP7 | ISM1 | FN1 |
|  | ANTXR1 | RAB31 | PXDN | DACT1 | PLXDC2 | HHIPL1 | C14orf37 |
|  | INHBA | KIF26B | SPARC | RUNX2 | PDLIM5 | ATXN1 | WISP1 |
|  | CDH11 | EDNRA | HMCN1 | SEPT11 | KIAA1217 | LUM | ITGBL1 |
|  | COL8A1 | TNFSF4 | SPOCK1 | TIMP2 | CTSK | FAM26E | FIBIN |
|  | COL5A2 | COL8A2 | PDGFRB | TIMP3 | PRSS23 | RASGRF2 | LOX |
|  | NTM | CTHRC1 | COL12A1 | COL1A2 | WNT2 | DKK3 | KCND2 |
|  | ITGA11 | CORIN | COL11A1 | CALU | MICAL2 | EVC | ASPN |
|  | ADAMTS12 | SULF1 | PRRX1 | ZNF281 | ADAM12 | SLC6A6 | ADAMTS2 |
|  | HSD17B6 | MXRA5 | COL3A1 | NUAK1 | FSTL1 | UNC5B | DCBLD1 |
|  | COL6A3 | ZFPM2 | FBN1 | TMEM200A | LTBP1 | TRIM59 | FAP |
|  | COL5A1 | KDELC1 | CHSY3 | PLS3 | GPX8 | GLT8D2 | CLIC4 |
|  | PDGFC | POSTN | FRMD6 |  |  |  |  |
| Only UALCAN | RP11-426C22.4 | RP11-752L20.3 | NREP | PRKG1 | AC093850.2 | AC004538.3 | CDKL5 |
|  | RP11-524D16__A.3 | AC106786.1 | METTL11B | PABPC4L | MSC-AS1 | PRDM6 | ZNF532 |
|  | SLC24A2 | PALLD | LOXL3 | HIP1 | PCDH7 | VGLL4 | SOCS5 |
| Only GEPIA | NOX4 | C5orf13 | C1QTNF3 | PCSK5 | PPAPDC1A | KAL1 | SCUBE2 |
|  | LRRC15 | HTRA1 | C3orf21 | CPZ | MYH9 | TPM4 | SSPN |
|  | COL1A1 | LRP1 | GPR161 | EIF5A2 | AEBP1 | FZD1 | MARVELD1 |

| Supplementary table 2: Functional enrichment analysis of COL10A1 and co-expressed genes for GO/KEGG in PAAD. | | | | | |
| --- | --- | --- | --- | --- | --- |
| GO | Category | Description | Count | Log(p) | Log(q) |
| GO:0001501 | GO Biological Processes | skeletal system development | 20/508 | -16.04 | -12.77 |
| GO:0001568 | GO Biological Processes | blood vessel development | 20/771 | -12.63 | -9.42 |
| GO:0070848 | GO Biological Processes | response to growth factor | 16/720 | -9.13 | -6.19 |
| GO:0007423 | GO Biological Processes | sensory organ development | 13/555 | -7.73 | -4.90 |
| GO:0007507 | GO Biological Processes | heart development | 13/595 | -7.38 | -4.56 |
| GO:0048729 | GO Biological Processes | tissue morphogenesis | 13/618 | -7.19 | -4.44 |
| GO:0032963 | GO Biological Processes | collagen metabolic process | 6/104 | -6.03 | -3.37 |
| GO:0031589 | GO Biological Processes | cell-substrate adhesion | 9/364 | -5.67 | -3.04 |
| GO:0060348 | GO Biological Processes | bone development | 7/206 | -5.41 | -2.81 |
| GO:0048568 | GO Biological Processes | embryonic organ development | 9/433 | -5.06 | -2.50 |
| GO:0085029 | GO Biological Processes | extracellular matrix assembly | 4/45 | -4.92 | -2.37 |
| GO:0048146 | GO Biological Processes | positive regulation of fibroblast proliferation | 4/48 | -4.80 | -2.27 |
| GO:2000027 | GO Biological Processes | regulation of animal organ morphogenesis | 5/125 | -4.33 | -1.87 |
| GO:0005604 | GO Cellular Components | basement membrane | 8/96 | -9.16 | -6.20 |
| GO:0005201 | GO Molecular Functions | extracellular matrix structural constituent | 23/172 | -30.79 | -26.44 |
| GO:0005518 | GO Molecular Functions | collagen binding | 8/69 | -10.33 | -7.28 |
| GO:0048407 | GO Molecular Functions | platelet-derived growth factor binding | 4/11 | -7.54 | -4.72 |
| GO:0005509 | GO Molecular Functions | calcium ion binding | 14/713 | -7.35 | -4.57 |
| GO:0002020 | GO Molecular Functions | protease binding | 6/135 | -5.37 | -2.78 |
| GO:0030021 | GO Molecular Functions | structural components of fibroblast proliferation | 3/22 | -4.36 | -1.882 |
| ko04974 | KEGG Pathway | Protein digestion and absorption | 8/90 | -9.38 | -6.63 |
| hsa04510 | KEGG Pathway | Focal adhesion | 7/199 | -5.51 | -3.21 |
| hsa05205 | KEGG Pathway | Proteoglycans in cancer | 5/218 | -3.21 | -1.26 |
| hsa05100 | KEGG Pathway | Bacterial invasion of epithelial cells | 3/85 | -2.62 | -0.72 |
| ko05146 | KEGG Pathway | Amoebiasis | 3/96 | -2.47 | -0.67 |
| hsa04060 | KEGG Pathway | Cytokine-cytokine receptor interaction | 4/270 | -1.99 | -0.37 |

Top 20 GO eichnrment analysis of COL10A1 and its co-expressed genes on biological processes, cellular components, and molecular functional categories, and top six KEGG pathway enrichment analysis. GO, Gene Ontology; KEGG, Kyoto Encyclopedia of Genes and Genomes.
