# Supplementary material for: Bioinformatics-Based Analysis: Noncoding RNA-Mediated COL10A1 Is Associated with Poor Prognosis and Immune Cell Infiltration in Pancreatic Cancer
Source: J Healthc Eng. 2022 Sep 5;2022:7904982. doi: 10.1155/2022/7904982 (PMC9467764; doi:10.1155/2022/7904982)
Supplement: Supplementary Materials — Supplement Figure 1: Functional Enrichment Analysis of Genes Coexpressed with COL10A1. Supplement Figure 2: Expression levels of COL10A1 in PAAD versus normal tissues from the GEPIA database. Supplement Figure 3: Ninety-six possible upstream lncRNAs predicted by StarBase. Supplementary Table 1: Intersection of the UALCAN database and the GEPIA database for coexpressed genes. Supplementary Table 2: Functional Enrichment Analysis of Genes Coexpressed with COL10A1. [file 7904982.f1.zip › 7904982.f1/Supplementary Table 1.docx]

| Supplementary table 1: Intersection of the UALCAN database and the GEPIA database for co-expressed genes. | | | | | | | |
| --- | --- | --- | --- | --- | --- | --- | --- |
| UALCAN AND GEPIA | THBS2 | VCAN | ST6GAL2 | ZFHX4 | FKBP7 | ISM1 | FN1 |
|  | ANTXR1 | RAB31 | PXDN | DACT1 | PLXDC2 | HHIPL1 | C14orf37 |
|  | INHBA | KIF26B | SPARC | RUNX2 | PDLIM5 | ATXN1 | WISP1 |
|  | CDH11 | EDNRA | HMCN1 | SEPT11 | KIAA1217 | LUM | ITGBL1 |
|  | COL8A1 | TNFSF4 | SPOCK1 | TIMP2 | CTSK | FAM26E | FIBIN |
|  | COL5A2 | COL8A2 | PDGFRB | TIMP3 | PRSS23 | RASGRF2 | LOX |
|  | NTM | CTHRC1 | COL12A1 | COL1A2 | WNT2 | DKK3 | KCND2 |
|  | ITGA11 | CORIN | COL11A1 | CALU | MICAL2 | EVC | ASPN |
|  | ADAMTS12 | SULF1 | PRRX1 | ZNF281 | ADAM12 | SLC6A6 | ADAMTS2 |
|  | HSD17B6 | MXRA5 | COL3A1 | NUAK1 | FSTL1 | UNC5B | DCBLD1 |
|  | COL6A3 | ZFPM2 | FBN1 | TMEM200A | LTBP1 | TRIM59 | FAP |
|  | COL5A1 | KDELC1 | CHSY3 | PLS3 | GPX8 | GLT8D2 | CLIC4 |
|  | PDGFC | POSTN | FRMD6 |  |  |  |  |
| Only UALCAN | RP11-426C22.4 | RP11-752L20.3 | NREP | PRKG1 | AC093850.2 | AC004538.3 | CDKL5 |
|  | RP11-524D16__A.3 | AC106786.1 | METTL11B | PABPC4L | MSC-AS1 | PRDM6 | ZNF532 |
|  | SLC24A2 | PALLD | LOXL3 | HIP1 | PCDH7 | VGLL4 | SOCS5 |
| Only GEPIA | NOX4 | C5orf13 | C1QTNF3 | PCSK5 | PPAPDC1A | KAL1 | SCUBE2 |
|  | LRRC15 | HTRA1 | C3orf21 | CPZ | MYH9 | TPM4 | SSPN |
|  | COL1A1 | LRP1 | GPR161 | EIF5A2 | AEBP1 | FZD1 | MARVELD1 |
