# Supplementary material for: Bioinformatics-Based Analysis: Noncoding RNA-Mediated COL10A1 Is Associated with Poor Prognosis and Immune Cell Infiltration in Pancreatic Cancer
Source: J Healthc Eng. 2022 Sep 5;2022:7904982. doi: 10.1155/2022/7904982 (PMC9467764; doi:10.1155/2022/7904982)
Supplement: Supplementary Materials — Supplement Figure 1: Functional Enrichment Analysis of Genes Coexpressed with COL10A1. Supplement Figure 2: Expression levels of COL10A1 in PAAD versus normal tissues from the GEPIA database. Supplement Figure 3: Ninety-six possible upstream lncRNAs predicted by StarBase. Supplementary Table 1: Intersection of the UALCAN database and the GEPIA database for coexpressed genes. Supplementary Table 2: Functional Enrichment Analysis of Genes Coexpressed with COL10A1. [file 7904982.f1.zip › 7904982.f1/Supplementary Table 2.docx]

| Supplementary table 2: Functional enrichment analysis of COL10A1 and co-expressed genes for GO/KEGG in PAAD. | | | | | |
| --- | --- | --- | --- | --- | --- |
| GO | Category | Description | Count | Log(p) | Log(q) |
| GO:0001501 | GO Biological Processes | skeletal system development | 20/508 | -16.04 | -12.77 |
| GO:0001568 | GO Biological Processes | blood vessel development | 20/771 | -12.63 | -9.42 |
| GO:0070848 | GO Biological Processes | response to growth factor | 16/720 | -9.13 | -6.19 |
| GO:0007423 | GO Biological Processes | sensory organ development | 13/555 | -7.73 | -4.90 |
| GO:0007507 | GO Biological Processes | heart development | 13/595 | -7.38 | -4.56 |
| GO:0048729 | GO Biological Processes | tissue morphogenesis | 13/618 | -7.19 | -4.44 |
| GO:0032963 | GO Biological Processes | collagen metabolic process | 6/104 | -6.03 | -3.37 |
| GO:0031589 | GO Biological Processes | cell-substrate adhesion | 9/364 | -5.67 | -3.04 |
| GO:0060348 | GO Biological Processes | bone development | 7/206 | -5.41 | -2.81 |
| GO:0048568 | GO Biological Processes | embryonic organ development | 9/433 | -5.06 | -2.50 |
| GO:0085029 | GO Biological Processes | extracellular matrix assembly | 4/45 | -4.92 | -2.37 |
| GO:0048146 | GO Biological Processes | positive regulation of fibroblast proliferation | 4/48 | -4.80 | -2.27 |
| GO:2000027 | GO Biological Processes | regulation of animal organ morphogenesis | 5/125 | -4.33 | -1.87 |
| GO:0005604 | GO Cellular Components | basement membrane | 8/96 | -9.16 | -6.20 |
| GO:0005201 | GO Molecular Functions | extracellular matrix structural constituent | 23/172 | -30.79 | -26.44 |
| GO:0005518 | GO Molecular Functions | collagen binding | 8/69 | -10.33 | -7.28 |
| GO:0048407 | GO Molecular Functions | platelet-derived growth factor binding | 4/11 | -7.54 | -4.72 |
| GO:0005509 | GO Molecular Functions | calcium ion binding | 14/713 | -7.35 | -4.57 |
| GO:0002020 | GO Molecular Functions | protease binding | 6/135 | -5.37 | -2.78 |
| GO:0030021 | GO Molecular Functions | structural components of fibroblast proliferation | 3/22 | -4.36 | -1.882 |
| ko04974 | KEGG Pathway | Protein digestion and absorption | 8/90 | -9.38 | -6.63 |
| hsa04510 | KEGG Pathway | Focal adhesion | 7/199 | -5.51 | -3.21 |
| hsa05205 | KEGG Pathway | Proteoglycans in cancer | 5/218 | -3.21 | -1.26 |
| hsa05100 | KEGG Pathway | Bacterial invasion of epithelial cells | 3/85 | -2.62 | -0.72 |
| ko05146 | KEGG Pathway | Amoebiasis | 3/96 | -2.47 | -0.67 |
| hsa04060 | KEGG Pathway | Cytokine-cytokine receptor interaction | 4/270 | -1.99 | -0.37 |

Top 20 GO eichnrment analysis of COL10A1 and its co-expressed genes on biological processes, cellular components, and molecular functional categories, and top six KEGG pathway enrichment analysis. GO, Gene Ontology; KEGG, Kyoto Encyclopedia of Genes and Genomes.
