# Supplementary figures and images for: Bioinformatics-Based Analysis: Noncoding RNA-Mediated COL10A1 Is Associated with Poor Prognosis and Immune Cell Infiltration in Pancreatic Cancer
Source: J Healthc Eng. 2022 Sep 5;2022:7904982. doi: 10.1155/2022/7904982 (PMC9467764; doi:10.1155/2022/7904982)

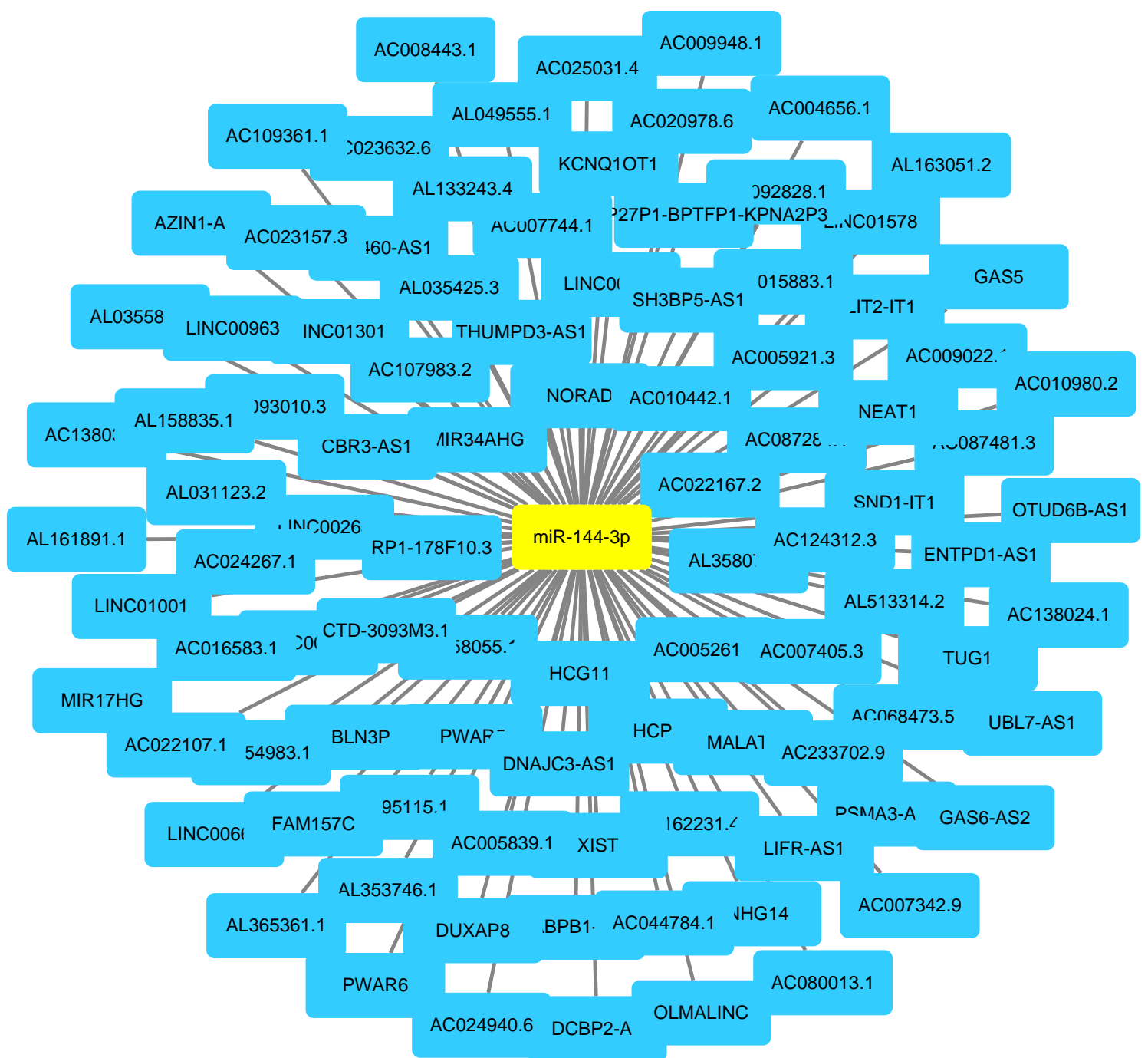

Supplement: Supplementary Materials — Supplement Figure 1: Functional Enrichment Analysis of Genes Coexpressed with COL10A1. Supplement Figure 2: Expression levels of COL10A1 in PAAD versus normal tissues from the GEPIA database. Supplement Figure 3: Ninety-six possible upstream lncRNAs predicted by StarBase. Supplementary Table 1: Intersection of the UALCAN database and the GEPIA database for coexpressed genes. Supplementary Table 2: Functional Enrichment Analysis of Genes Coexpressed with COL10A1. [file 7904982.f1.zip › 7904982.f1/Supplement Figure.3.pdf]

COL10A1

Expression  $\lg_2(\text{TPM} + 1)$

10  
8  
6  
4  
2  
0

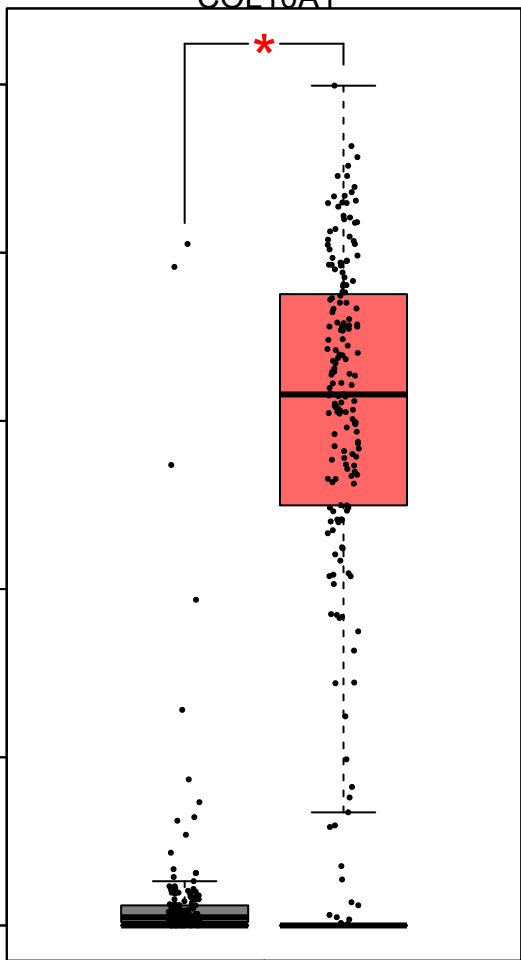

Normal

PAAD

(num(T)=179; num(N)=171)

Supplement: Supplementary Materials — Supplement Figure 1: Functional Enrichment Analysis of Genes Coexpressed with COL10A1. Supplement Figure 2: Expression levels of COL10A1 in PAAD versus normal tissues from the GEPIA database. Supplement Figure 3: Ninety-six possible upstream lncRNAs predicted by StarBase. Supplementary Table 1: Intersection of the UALCAN database and the GEPIA database for coexpressed genes. Supplementary Table 2: Functional Enrichment Analysis of Genes Coexpressed with COL10A1. [file 7904982.f1.zip › 7904982.f1/Supplementary Figure.2.pdf]
